# PoulTrans: a transformer-based model for accurate poultry condition assessment

**DOI:** 10.1038/s41598-025-98078-w

**Published:** 2025-04-23

**Authors:** Jun Li, Bing Yang, Junyang Chen, Jiaxin Liu, Felix Kwame Amevor, Guanyu Chen, Buyuan Zhang, Xiaoling Zhao

**Affiliations:** 1 College of Information Engineering, Sichuan Agricultural University, 46 Xinkang Road, Yucheng District, Ya’an, 625000 Sichuan Province People’s Republic of China; 2 Agricultural Information Engineering Higher Institution Key Laboratory of Sichuan Province, Ya’an, 625000 Sichuan Province People’s Republic of China; 3 Ya’an Digital Agricultural Engineering Technology Research Center, Ya’an, 625000 Sichuan Province People’s Republic of China; 4 Key Laboratory of Livestock and Poultry Multi-Omics, College of Animal Science and Technology, Sichuan Agricultural University, Chengdu, 611130 Sichuan People’s Republic of China

**Keywords:** Deep learning, Poultry state, Image caption, Transformer, Poultrans, Zoology, Engineering

## Abstract

Recent advances in deep learning have significantly enhanced the accuracy of poultry image recognition, particularly in assessing poultry conditions. However, developing intuitive decision support tools remain a significant challenge. To address this, we present PoulTrans, an innovative image captioning framework that leverages a Convolutional Neural Network (CNN) integrated with a CSA_Encoder-Transformer architecture to generate detailed poultry status reports. This model incorporates visual features extracted by CNNs into the Channel Spatial Attention Segmentation Encoder (CSA_Encoder), which produces segmented channel and spatial attention outputs. To optimize multi-level attention and improve the semantic precision of the status descriptions, we introduced a Channel Spatial Memory-Guided Transformer (CSMT) and a novel PS-Loss function. The performance of PoulTrans was tested on the PSC-Captions dataset, achieving top scores of 0.501, 0.803, 4.927, 0.608, and 1.882 for the BLEU-4, ROUGE-L, CIDEr, SPICE, and Sm metrics, respectively. Comprehensive analyses and experiments have validated the effectiveness and reliability of our model, providing advanced tools for automated poultry status generation and enhancing the digital experience for poultry farmers. Our code is available at: https://github.com/kong1107800/PoulTrans.

## Introduction

Poultry is a critical component of human diet, serving as a major source of animal protein worldwide. According to the Food and Agriculture Organization of the United Nations (FAO), poultry products rank as the most consumed source of animal protein globally^1^. As the population continues to grow, so does the demand for poultry products, driving further expansion of the poultry industry. Accurate monitoring of poultry health and condition is essential to ensure their well-being, as poor conditions can lead to diseases, which in turn can significantly reduce production in the global poultry industry and potentially pose risks to human health^2,3^. Initially, human workers were tasked with manually analyzing poultry status but this process is time-consuming^4^.

Deep learning, a key branch of machine learning, leverages hierarchical learning and abstraction to extract deep features from data using multi-layer network architectures^5^. This approach has proven to be a powerful tool for addressing complex problems in computer vision^6^. Since the introduction of neural network-based backpropagation algorithms by Rumelhart et al. in 1986, there has been a significant advancement in the research of deep neural networks^7^. Notably, in 1989, LeCun et al. introduced the LeNet model^8^ designed for two-dimensional image variability, which drove the early development of convolutional neural networks. By 2017, the Google research team introduced the Transformer network architecture, which achieved unprecedented success in machine translation^9^, profoundly impacting natural language processing and becoming a standard model for core tasks in the field^10^.In recent years, researchers have explored the application of deep learning techniques to the challenging task of assessing poultry condition to achieve significant results. For instance, Huang et al. developed an audio-analysis-based method for detecting poultry influenza by training an SVM classifier to recognize chicken cries, with an accuracy of 84% to 90%^11^. Cuan et al. proposed a convolutional neural network (CSCNN) for detecting poultry influenza using chicken cries to achieve promising experimental results^12^. Okinda et al.^14^ developed a machine vision-based system to monitor poultry movement, feature variables using 2D pose descriptors and motion characteristics for non-invasive disease prediction^13^. Degu et al. used the YOLOv3 target detection algorithm and ResNet50 image classification model to create a system to identify poultry diseases with 87.48% detection accuracy and 98.7% classification accuracy for three major poultry diseases.

However, current studies are primarily focused on traditional deep learning methods that heavily rely on unimodal data extraction, such as visual or auditory features. In the visual domain, studies often emphasize pixel-level or object-level data interpretation, overlooking the importance of contextual information^15^. In the auditory domain, researchers face challenge in noise suppression due to varying background noise in different environments. Crucially, these unimodal methods do not link state results to their underlying basis, providing only state results the reasoning behind them, which limits their usefulness to breeders. Although existing research has attempted to address this challenge, it still faces three central problems:

1. How to generate a comprehensive poultry status report that includes all types of poultry status?
2. How to ensure that semantic information in poultry status reports is actually useful to farmers?
3. How to achieve a differentiated descriptive focus for features of different states?

To address the first challenge, we developed a poultry state report generation model based on CNN-CSAEncoder-Transformer architecture and constructed the first dataset PSC-Captions dedicated to poultry state reports. The model can efficiently learn the state features of poultry from the PSC-Captions dataset and distinguishes.

To address the second challenge, the PSC-Captions dataset contains detailed poultry condition information such as movement posture, health status and disease type. In the case of disease, the report also includes information on disease symptoms, providing farmers with comprehensive status information.

To overcome the third difficulty, we designed the CSA-Encoder to channelize and spatially process the features extracted by the CNN and feed them into the innovative CSMTransformer text decoder. This ensures the accuracy of the state descriptions and makes the descriptions more realistic and effective in different states.

The main highlights of this study can be summarized as follows:

1. The first image caption task dataset, PSC-Captions, was constructed specifically to characterize poultry conditions.
2. A novel multi-attention encoder proposed to optimize the calibration of diverse state features.
3. Development of a multilevel attentional memory text decoder to achieve persistent memory of linguistic information features.
4. Design the enhanced loss function PS-Loss and introduce the concept of comparative learning to improve the model performance.
5. Developed a report generation model based on the CNN-CSA_Encoder-Transformer architecture, demonstrated excellent experimental results through a series of experiments, and verified its effectiveness and generalization ability.
6. Design and propose "Weighted Evaluation Indicators" (Sm) to provide new quantitative criteria for a comprehensive assessment of the quality of poultry status reports.

This manuscript is organized as follows: Chapter 2 details the key techniques of the PoulTrans model. Chapter 3 validates the model’s performance through experiments and explores its internal mechanisms. Chapter 4 summarizes the results of this study and its potential application to poultry status reporting, and the final chapter discusses research limitations and future research directions.

## Method

### General

The baseline model in this study utilizes the popular "encoder-decoder" architecture initially introduced by Vinyals et al^16^. Poultrans employs a three-stage architecture, with ResNeXt101 which serve as the image feature extractor. It incorporates a multi-attention feature differentiation encoder called CSA-Encoder, and a CSMTransformer (CSMT) for text decoding. The architecture is specifically designed to process and analyze image features from various poultry states (with different feature locations) to generate precise reports on poultry status. In addition, to enhance the extraction of information between text and image patterns, we introduced the PS-Loss contrast learning loss function.

**Fig. 1:**
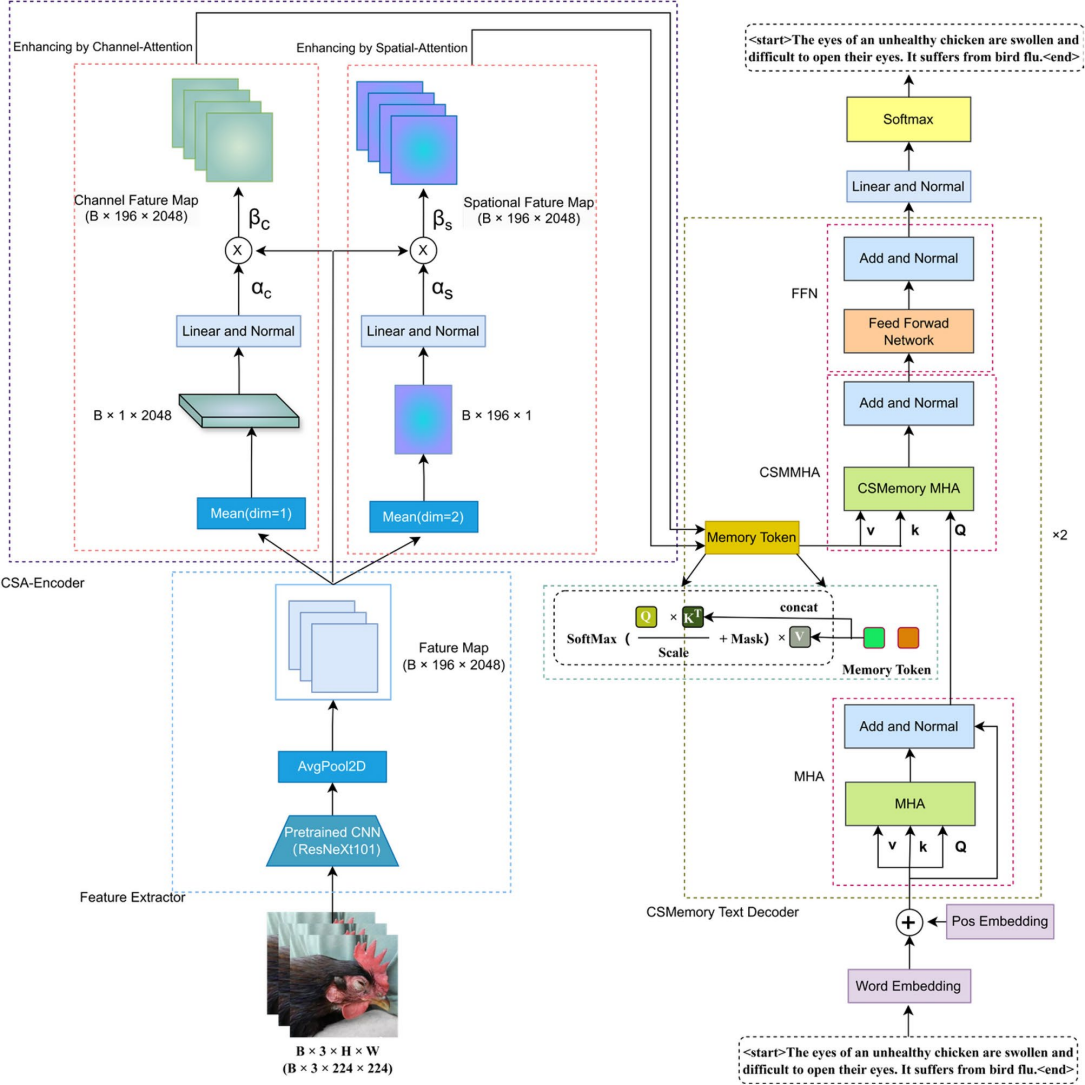
Schematic diagram of PoulTrans network structure.

The workflow of the PoulTrans model is shown in Fig. 1. The model first receives N poultry state images from the training set as input I = {Ii |0 ≤ i ≤ N}. These images are processed by the ResNeXt101 image feature extractor, which utilizes its grouped residual structure to efficiently extract high-level feature maps in the images F1 ∈ ℝW × H × C. Subsequently, these high-level features are fed to the CSA-Encoder multi-attention feature encoder. This encoder enhances the convolutional feature map with dynamic attention to channel and spatial information and maps its size back to the size of F1 uniformly. The encoder outputs the enhanced channel attention and spatial attention feature maps F2 ∈ ℝ2W × H × C, where W, H, and C represent the width, height, and channel depth, respectively. After that, the enhanced channel attention and spatial attention feature maps are fed to the CSMT text decoder as V and K, respectively. During decoding, a dynamic memory unit enhances the model’s understanding of the state information and the connections between feature within the state image. The decoder integrates the enhanced attention features with historical data stored in the memory unit, to allow for a more in-depth analysis of the current image content using the multi-head attention mechanism. Finally, the model transforms the synthesized features into a final state report Di ∈ ℝ3B × S × V through the feed-forward network layer. where S denotes the maximum sequence output length and V represents the vocabulary size. Efficient ResNeXt101 Image Feature Extractor.

In the feature extraction phase, we followed the approach reported by Yang’s^17^ and chose the ResNeXt101 model pre-trained on ImageNet^18^ to extract image features. This approach can speed up the convergence of the model and learn effective image features faster. Since the goal of this stage was to extract image features, we discarded the final pooling and linear layers of the original ResNeXt and fine-tune some of the weights of ResNeXt101.

**Fig. 2:**
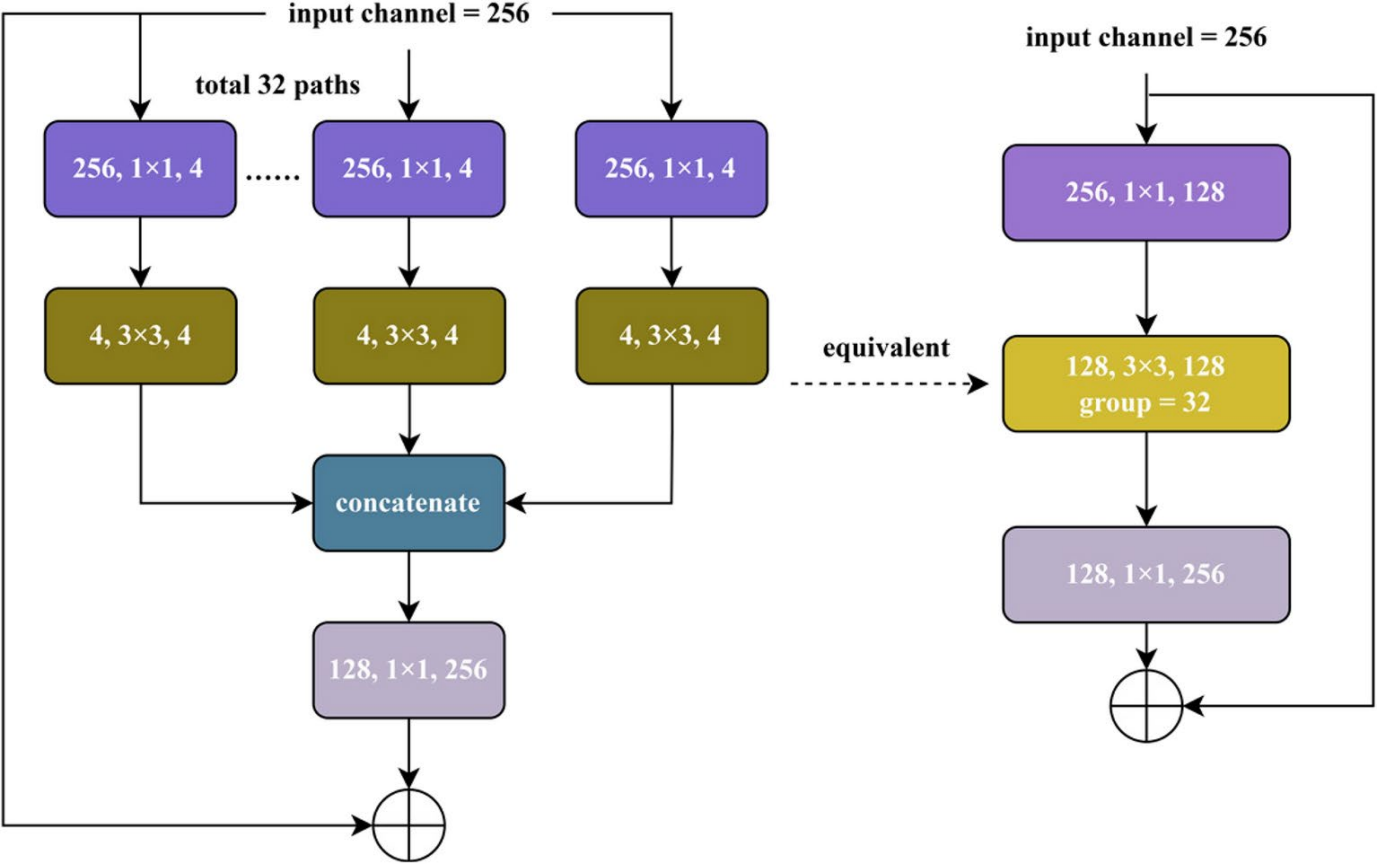
Schematic diagram of ResNeXt101 image feature extractor.

ResNeXt101, as an improved version of the ResNet architecture, introduces the concept of “cardinality”, thus, the number of paths, to enhance the expressive power and computational efficiency of the network. Instead of directly increasing the size of the convolutional kernel or the number of filters, ResNeXt101 increases the number of grouped convolutions to improve the model performance while effectively controlling the growth of the number of parameters. The size of the input image is 3*224*224, and the output is batchsize × 196 × 2048. Specifically, the model compresses the data with 256 input channels into 32 groups by 1 × 1 convolution, with 4 channels in each group, totaling 128 channels. After the convolution operation, the data is expanded back to 256 channels by 1 × 1 convolution, and finally the 32 groups of data are summed at the corresponding positions to generate a 256 channels output. The framework of the feature extractor is shown in Fig. 2.

### Multi-attention visual feature differentiation encoders

**Fig. 3:**
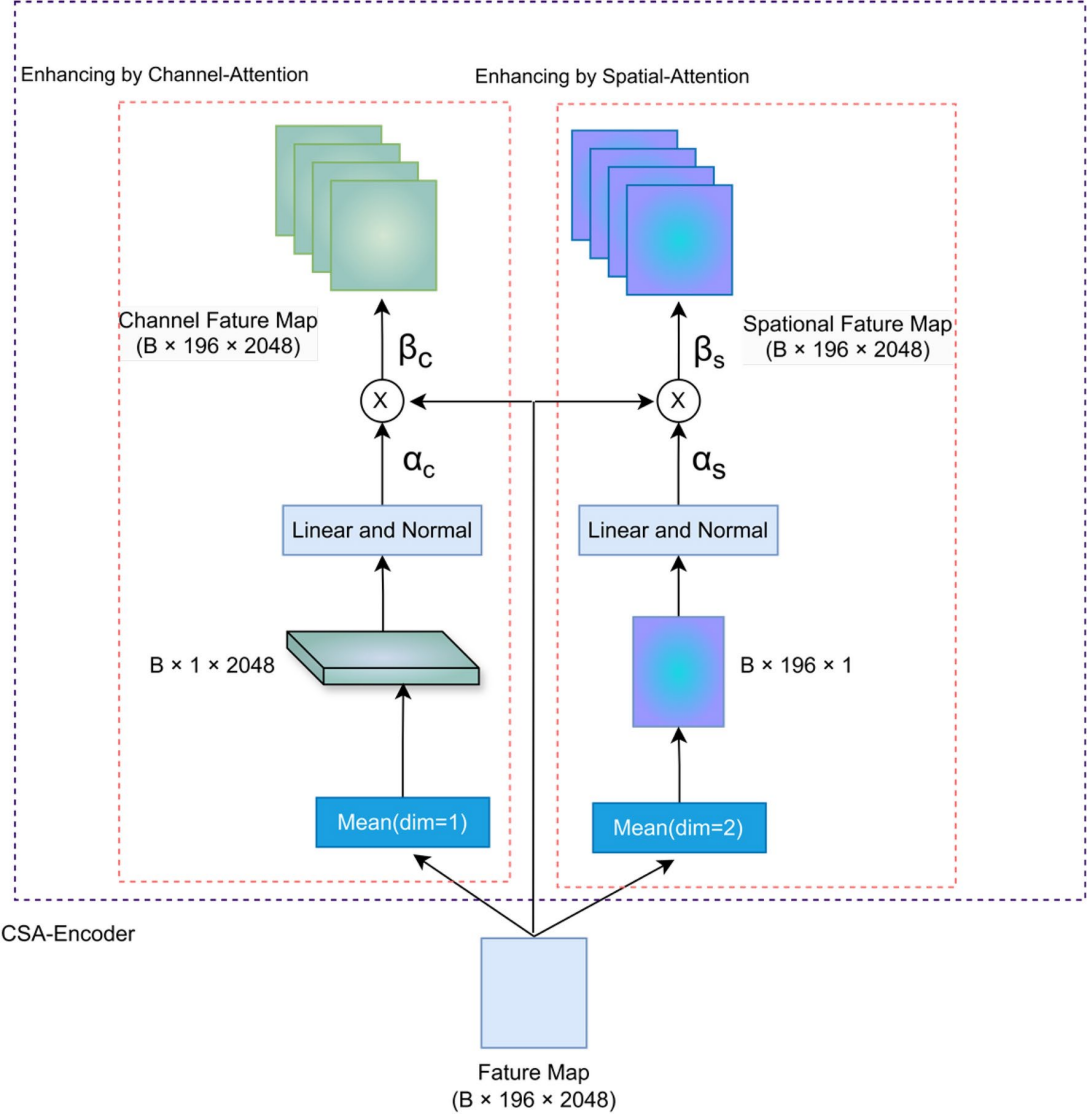
Multi-attention visual feature differentiation encoders architecture.

To overcome the limitation of visual feature extractors, which was unable to dynamically select different state feature regions to generate specific sentences while capturing rich semantic information in state images, therefore, this study introduces a multi-attention encoder. The encoder extracts hierarchical attentional features by identifying multiple key state feature regions within the poultry state image, thereby enhancing the model’s understanding of the state image. Specifically, the model processes the high-level semantic feature map F1 extracted by the visual feature extractor, and then apply channel and spatial attention to it, as shown in the architectural diagram (Fig. 3).

### Channel attention characterization

The channel attention mechanism was concerned with the recognition of each semantic attribute and the learning of the scale variations of the different state feature regions within the image of the poultry class. The channel attention weights were generated by synthesizing the visual feature map F1 in order to retrieve channel descriptors and achieve scale diversity through weight parameters. These channel attention weights refine the global feature map in order to produce the channel attention feature map (β*c*). The mathematical expression for this process is provided below:

Channel Average Pooling: designed to capture the global response of each channel and encode global statistics for each channel.

1 $${c}_{\text{avg }}=\frac{1}{C}\sum_{c=1}^{C} {x}_{fc}^{c}$$

where C is the number of channels and ${x}_{fc}^{c}$ denotes the cth channel.

Learning channel weights: capturing inter-channel dependencies by adaptively learning the importance of different channels through the weight parameter.

2 $${W}_{c}=\sigma \left({W}_{c\text{ \_params }}\cdot {c}_{avg}\right)$$

${W}_{c\text{ \_params}}$ represents the learning parameters of the channel attention, and $\sigma$ represents the sigmoid activation function.

Generating channel attention features: enhancing differentiation of lesion feature representations by emphasizing important channels.

3 $${\beta }_{c}={x}_{fc}\cdot {W}_{c}$$

The channel attention mechanism enables the model to adaptively adjust the feature responses of different channels, thereby emphasizing those channels that are more pertinent to the current state judgement while suppressing those that are less so. This mechanism facilitates the effective differentiation of different state categories and their feature complexity when recognizing poultry state features.

### Spatial attention characterization

The objective of the spatial attention mechanism is to enhance the model’s capacity to recognize state feature regions. By aggregating the global feature map in the spatial dimension, a single feature map is obtained which synthesizes the overall spatial information (${S}_{avg}$). The aforementioned feature map is subsequently employed to generate spatial attention weights through the learning of weight parameters, thereby facilitating the recognition of the location diversity of state feature regions. These weights were then utilized to highlight the importance of state feature regions, thus, providing a more accurate characterization of spatial attention (${\beta }_{s}$). The mathematical formulation of the process is as follows:

Spatial aggregation of global feature maps: The global statistical information of the feature maps is encoded by averaging the aggregation along the spatial dimension for each feature channel.

4 $${S}_{avg}=\frac{1}{H\times W}\sum_{h=1}^{H} \sum_{w=1}^{W} {x}_{fc}\left(h,w\right)$$

where H and W are the height and width of the feature map, respectively. ${x}_{fc}\left(h,w\right)$ denotes the feature vector at position $\left(h,w\right)$ of the feature vector.

Learning spatial location diversity: capturing lesion location diversity by adaptively learning the importance of different spatial locations on the feature map through weighting parameters.

5 $${W}_{s}=\sigma \left({W}_{\text{s\_params }}\cdot {S}_{\text{avg}}\right)$$

where ${W}_{\text{s\_params}}$ represents the attention weights, and $\sigma$ represents the sigmoid activation function.

Generating spatial attention features: further enhancing the characterization of features by focusing on important spatial regions, i.e., state feature regions.

6 $${\beta }_{s}={x}_{fc}\cdot {W}_{s}$$

The spatial attention mechanism enables the model to effectively aggregate the global feature map in a spatially aware manner, thereby facilitating the adaptive learning and emphasizing of the spatial locations of state feature regions. This results in a notable enhancement in the model’s ability to characterize state features. Furthermore, the accuracy in recognizing state feature regions has been augmented.

### Multi-attention based memory feature-guided decoder

**Fig. 4:**
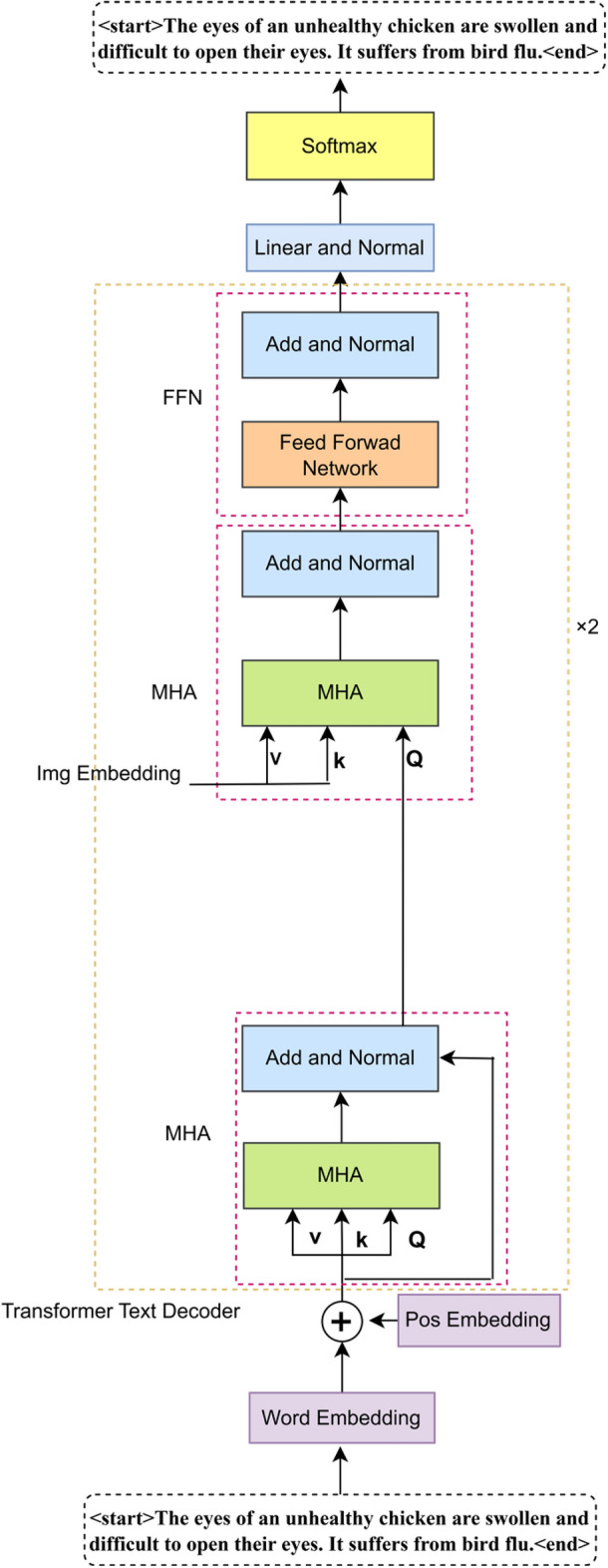
Schematic diagram of Transformer.

In order to fully utilize the multiple attentional feature maps extracted by the encoder, this paper presents a text decoder based on the guidance of multiple attentional memory features, as illustrated in Fig. 4. The objective of the decoder is to integrate visual features (including channel and spatial features) with linguistic information in order to generate text descriptions that are both accurate and semantically rich. The proposed decoder is based on the classical Transformer framework, which comprises a stack of two multi-layer attention memory-guided Transformer layers. The key distinction lies in the addition of a layer of multi-head self-attention, which is employed for computing the attention score of each word. The CSMemory-MultiHead-Attention layer replaces the standard MultiHead-Attention layer and combines PointWiseFeedForwardNetwork, LayerNorm and dropout techniques to enhance the model’s generalization and learning efficiency. The overall process is as follows:

The differentiated spatial and channel attentions connect memory units as K and V of the memory polytope self-attention, respectively, and the self-attention of word vectors is computed as Q of the memory polytope self-attention:

7 $${K}_{\text{final }}=\text{cat}\left(k,{W}_{k}\cdot {K}_{\text{init }},\text{dim}=-1\right)$$

8 $${V}_{\text{final }}=\text{cat}\left(v,{W}_{v}\cdot {V}_{\text{init }},\text{dim}=-1\right)$$

9 $${Q}_{\text{final }}=\text{AddNorm}(\text{MultiHead }\left.\left({S}_{i}^{\text{in}},{S}_{i}^{\text{in}},{S}_{i}^{\text{in}}\right)\right)$$

Because of the stack of 2 Transformer layers, the output of the previous attention block is the input of the next attention block. Let Ft_i_ be the output of the last attention block. Finally, the description decoder uses a connected layer to transform Ft_i_ to create a probability distribution for each probability.

10 $$F{t}_{i}^{1}=\text{AddNorm}\left(FFN\left(\text{AddNorm}(\text{MultiHead }\left.\left({K}_{\text{final }},{V}_{\text{final }},{Q}_{\text{final}}\right)\right)\right)\right)$$

**Fig. 5:**
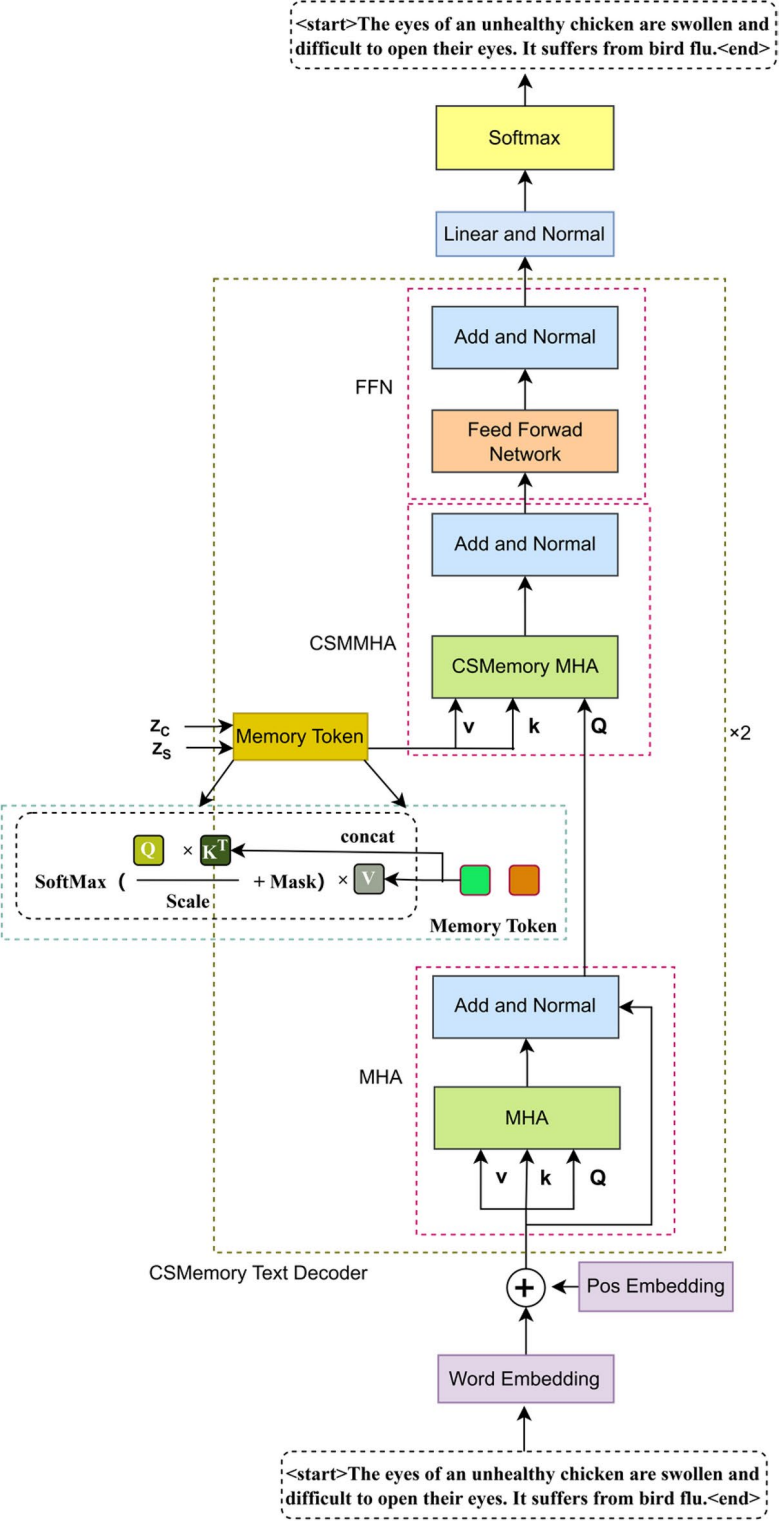
Schematic diagram of CSMT.

For comparison, we also show the structure of the standard Transformer and the Multi-Attention Memory Transformer in Figs. 4 and 5.

### Loss function

In recent years, the design of loss function has proven to be crucial for enhancing model performance in image captioning tasks^19–21^. Traditionally, many studies have relied on a simple Cross Entropy Loss to measure the difference between the model-generated text and the target text. However, this method fails to fully utilize the correlation between image and text representations, limiting the model’s ability to produce accurate and semantically rich poultry status reports. To address this issue, we introduced a novel and powerful loss function, PS-Loss (Poultry State Loss), which improves performance by accounting for the similarity between images and text. PS-Loss is comprised of two components: an image mapping loss and a text cross-entropy loss. The objective of the image mapping loss is to map image features to a new feature space through a linear transformation, thereby making the image features in this space more semantically similar to the corresponding text features. In particular, the image channel features are initially mapped from 2048 to 1198 dimensions through a linear layer. Subsequently, the image and text alignment are achieved by randomly selecting a portion of the mapped features, and the cosine similarity is calculated with all the text features to form a similarity matrix. Let Imapped be the mapped image feature vector and T be the text feature vector. Then, the image mapping loss is defined as:

11 $${\text{I}}_{\text{mapped}}=\text{W}\cdot \text{I}+\text{b}$$

where W and b denote the weights and bias terms of the linear layer, respectively, and I is the original image feature vector. Through this mapping, the cosine similarity S between I_mapped_ and T is calculated to form a similarity matrix. Based on this, the image mapping loss calculation formula is further refined as:

12 $${\text{Loss}}_{\text{image}}=\frac{1}{\text{N}}\sum_{\text{i}=1}^{\text{N}}\left(1-\text{diag}\left({\text{S}}_{\text{i}}\right)+\text{max}\left(0,\text{ S}-\text{m}\right)\right)$$

In this context, S represents the similarity matrix, Si represents the ith element of the similarity matrix, and diag (Si) denotes the similarity values between each image feature vector and its corresponding text feature vector, extracted from the main diagonal of the matrix. The similarity matrix Sm is a predefined margin value, which was set to 0.2 in the experiments. This margin controls the difference between positive and negative samples, to help the model generate image features that are more closely align with the correct text.

In contrast, the text cross-entropy loss component quantifies the degree of alignment between the text sequence generated by the model and the target text sequence, providing a direct assessment of the quality of text generation. Let the predicted probability distribution of the model for each word be P, while the real word distribution is Q, then the text cross-entropy loss is defined as follows:

13 $${\text{Loss}}_{\text{text}}=-\sum_{\text{i}}{\text{Q}}_{\text{i}}\text{log}\left({\text{P}}_{\text{i}}\right)$$

Here, the index of the entire vocabulary is traversed, and the formula facilitates the generation of text sequences that are both syntactically and semantically more accurate by reducing the discrepancy between the predicted and true distributions.

The combination of image mapping loss and text cross-entropy loss, which we refer to as PS-Loss, effectively guides the model to generate image features that are more similar to the correct text while ensuring text sequence accuracy. Additionally, learnable loss weights dynamically balance the contributions of these losses, providing flexibility to adapt to different tasks and dataset requirements. In order to calculate the total loss L, we first define the image mapping loss weight ${\uplambda }_{\text{image}}$ and the text cross-entropy loss weight ${\uplambda }_{\text{text}}$. Then, we have:

14 $$\text{L}={\uplambda }_{\text{image}}\cdot {\text{Loss}}_{\text{image}}+{\uplambda }_{\text{text}}\cdot {\text{Loss}}_{\text{text}}$$

Among the formulas, both λ_image_ and λ_text_ are set to 0.5 in the initialization phase and can be automatically optimized by backpropagation during the training process to balance the impact of the two losses on the model performance.

## Experimentation

### Introduction to the datasets

The basic information of PSC-Captions and Flickr8k is shown in Table 1.

**Table 1:** Feature comparison between the two datasets of PSC-Captions and Flickr8k.

Dataset	Total-images	Total-captions	Image-size	Image-type	Train	Val
PSC-Captions	8294	33176	224 × 224	JPEG	6859	1435
Flickr8k	8090	40,450	224 × 224	JPEG	6472	1618

### PSC-Captions (poultry state characteristics)

The PSC-Captions dataset presented in this study was the first comprehensive effort to compile and integrate data specifically designed to describe the condition of birds. The term “poultry” encompasses domesticated birds, with chickens being the most raised worldwide, followed by ducks and geese^22^. Due to the significant similarities in appearance and condition among various poultry species, four representative species chickens, ducks, geese, and pigeons were selected to construct this poultry condition image dataset.

**Fig. 6:**
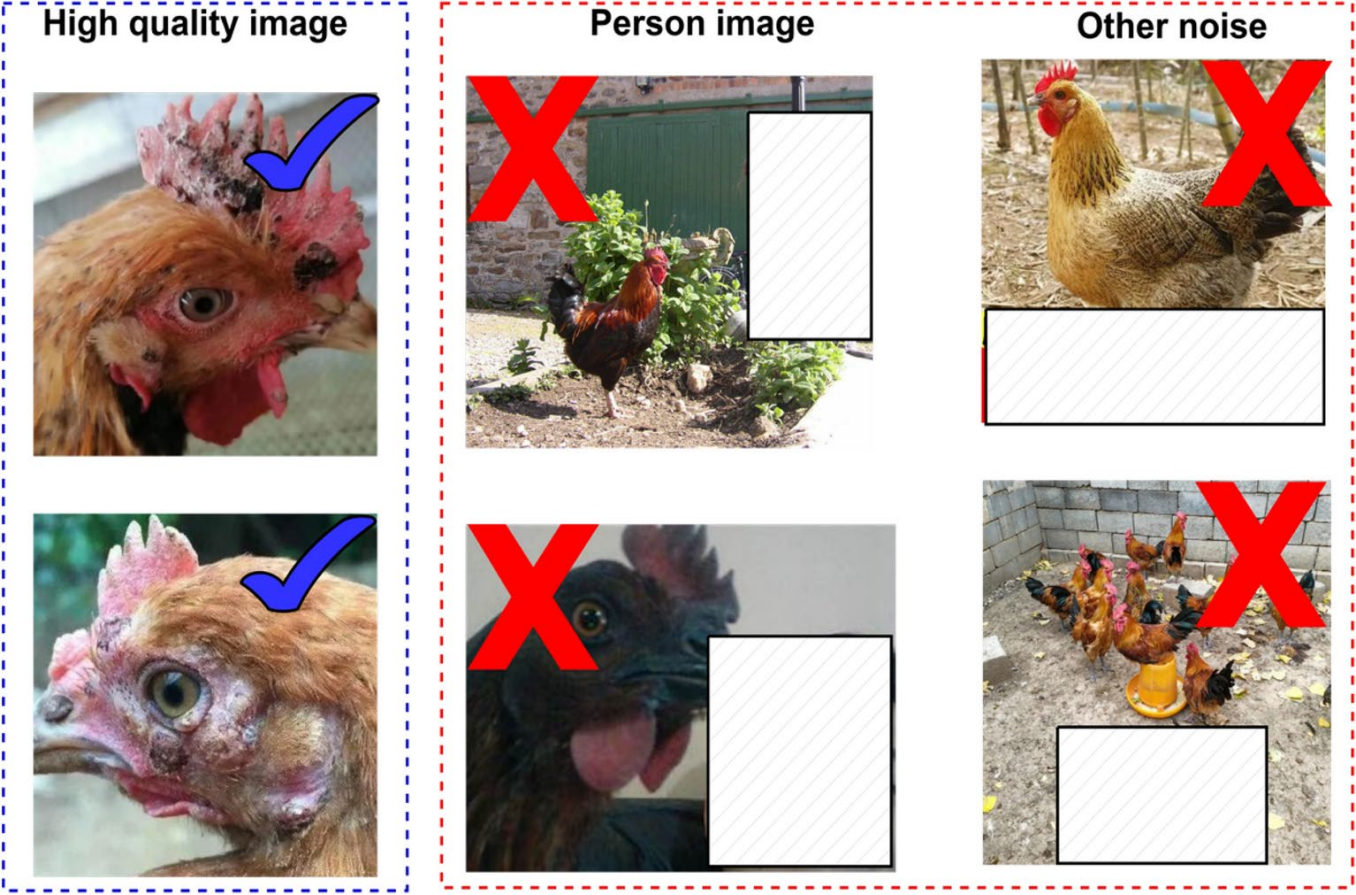
Examples of high-quality images and noisy images.

To ensure the applicability of the dataset across diverse agricultural settings, a total of 10,000 potential images were sourced through a combination of on-farm photography and web crawling, with the consent of local farms and in compliance with relevant regulations. Images that were not displayable were removed, and all remaining images were converted to JPEG format. Images with a resolution of less than 224 × 224 were filtered out and adjusted to a uniform resolution of 224 × 224. In the case of images with significant noise and ambiguity, the dataset was filtered to exclude images containing characters using the pre-trained YOLOv5 model. The examples of high-quality images and noisy images are shown in Fig. 6. With the help of agricultural experts, the images were filtered in this final step, resulting in a total of 7179 images.

**Fig. 7:**
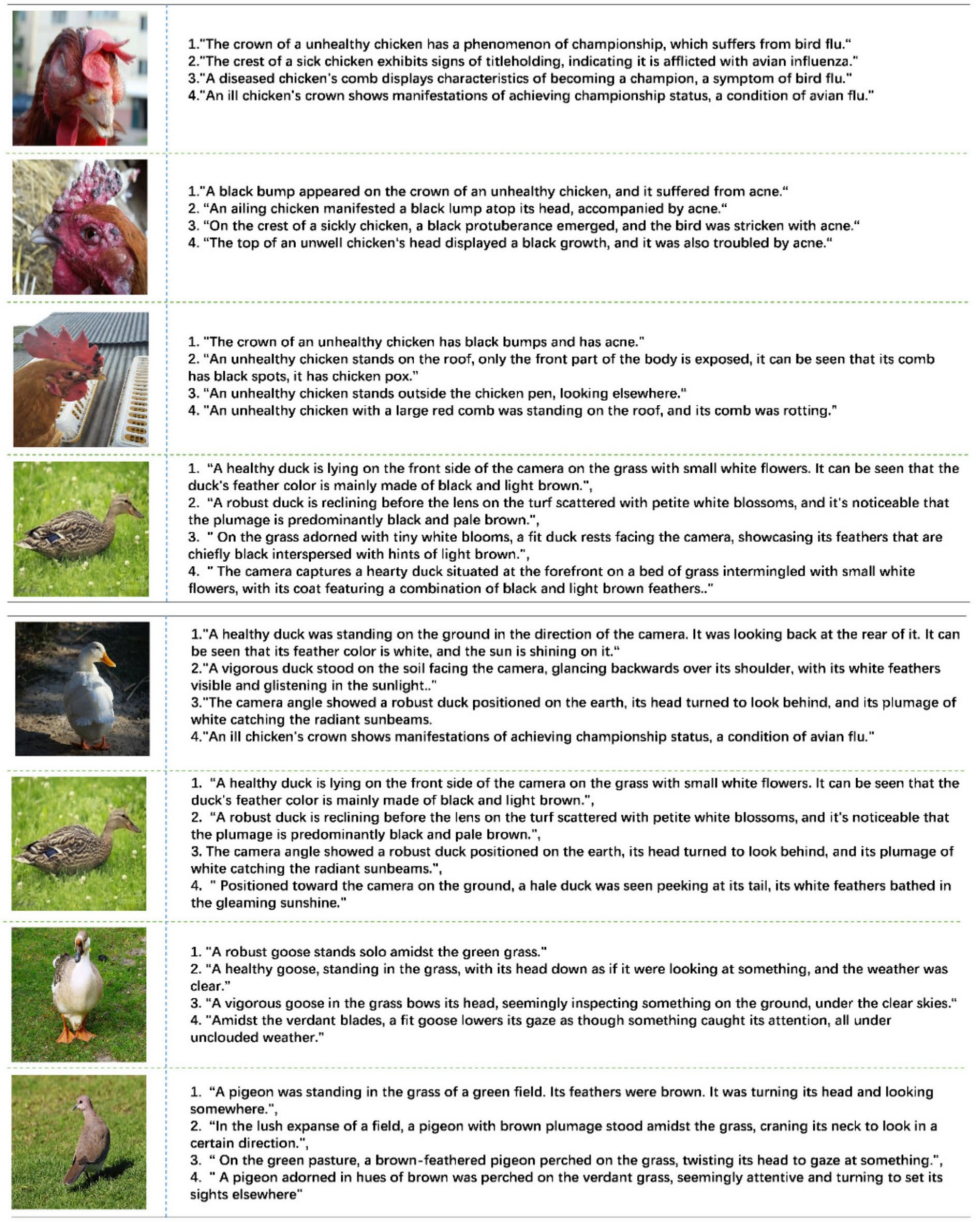
Some examples from PSC-Captions.

The condition descriptions followed the COCO-Captions format^23^. The image dataset was randomly perturbed and four agricultural experts were asked to describe the condition of 5179 poultry images. To minimize researcher-induced subjectivity, the remaining 2000 images were annotated using the pre-trained BLIP model^24^, with expert review and necessary corrections. To prevent information loss due to single state description, artificial noise, such as variations in feather color and environmental factors, was incorporated into the final dataset. An example of the PSC-Captions dataset is presented in Fig. 7.

### Flickr8k dataset

**Fig. 8:**
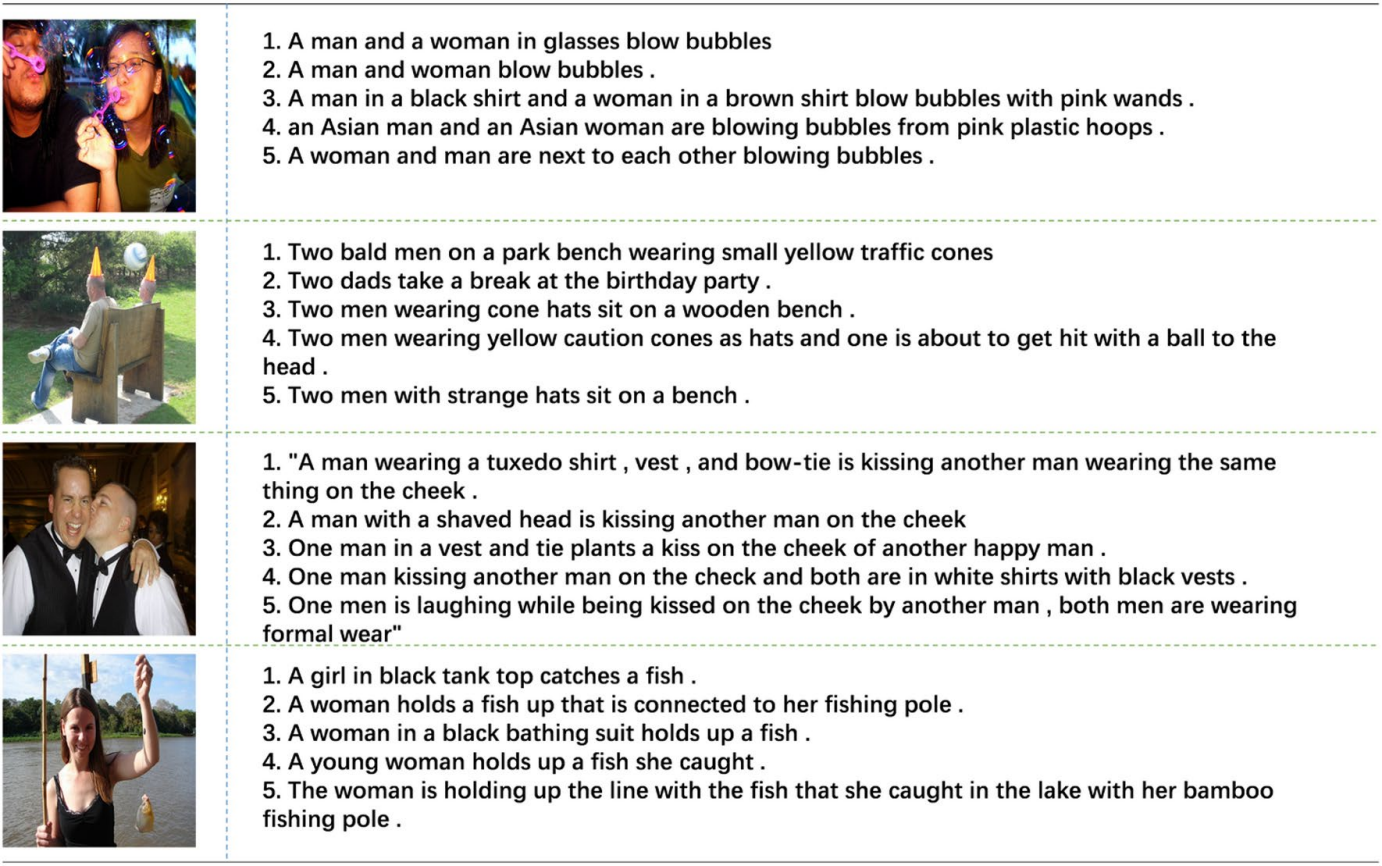
Some samples from Flickr8k.

The Flickr8k dataset is a key benchmark for image caption generation which contains over 8000 images of various everyday situations, such as personal activities, human interactions, indoor scenes and outdoor environments, each accompanied by five descriptions from different annotators. A sample of the Flickr8k dataset is shown in Fig. 8.

### Preprocessing of datasets

**Fig. 9:**
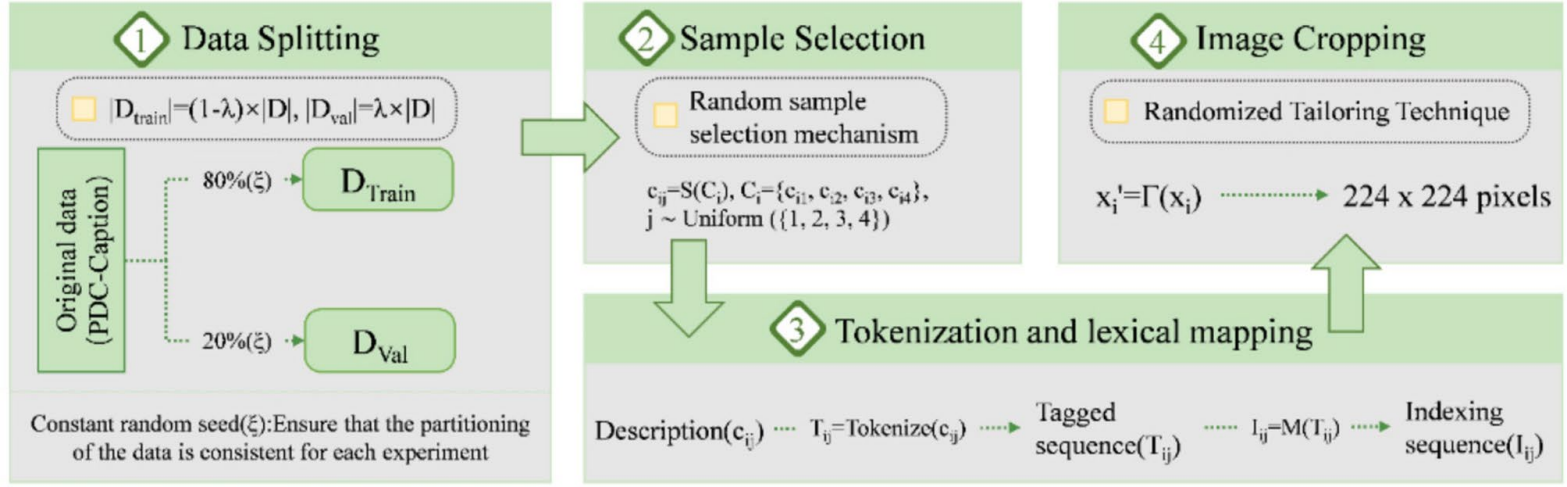
Illustrates the flowchart of the dataset preprocessing.

As illustrated in Fig. 9, the PSC-Captions dataset was subjected to preprocessing in the course of this study with a view to guaranteeing the validity of the model training and the reproducibility of the experimental results.

*Dataset Preparation*: The dataset was divided into training (${D}_{\text{train}}$) and validation (${D}_{\text{val}}$) sets in an 8:2 ratio. Each image (${x}_{i}$) and its description set (${C}_{i}$) had one randomly selected description (${c}_{ij}$) tokenized into sequence (${T}_{ij}$). Frequent words (occurrence ≥ 2) were indexed via lexical mapping *M*.

*Image Preprocessing*: During training and validation, images were randomly cropped and resized to 224 × 224 pixels to enhance model adaptability.

### Experimental equipment

The Poultrans comparison and ablation experiments were conducted on the identical experimental platform and utilized the same parameters, as illustrated in Table 2.

**Table 2:** Experimental platform and parameter settings.

Parameter	Value	Parameter	Value
Epoch	200	Optimizer	Adam^25^
Embedding	1024	Optimizer Alpha	0.9
Decoder Heads	8	Optimizer Beta	0.999
Decoder Layers	2	Encoder_lr	1e–4
Batch Size	16	Decoder_lr	5e–5
Learning Rate Annealing	Every 3 cycles	Annealing Rate	0.8x
Pytorch	2.2.2	Early Stop	50
OS	Windows	CPU	Intel 6230R
GPU	RTX 4090	Software	Python 3.8

### Evaluation indicators

To comprehensively evaluate the quality of the generated poultry status reports, several evaluation metrics were used, including BLEU^26^, ROUGE^27^, CIDEr^28^, SPICE^29^ and Sm. BLEU is a metric that assesses the accuracy of the literal content, ROUGE measures the breadth of information covered, CIDEr evaluates the contextual relevance of the text, and SPICE analyses the semantic components (e.g. objects, attributes, relations, etc.) to assess the semantic accuracy of the generated text.

SPICE emphasizes semantic precision and completeness, ROUGE focuses on key information coverage, and CIDEr on contextual relevance. Together, these three metrics provide a comprehensive framework for condition assessment. Meanwhile, the BLEU metrics, while primarily assessing the literal accuracy of reports, still play an important role in the overall evaluation system. In view of this, we combine the characteristics of these existing assessment metrics and propose a comprehensive assessment metric, Sm, specifically for the generation of poultry status reports. The aim is to quantitatively measure the overall performance of the poultry status report generation model. The formula for Sm is as follows:

15 $${S}_{m}=\frac{1}{7}BLE{U}_{4}+\frac{2}{7}\left({ \text{ROUGE }}_{L}+SPICE+CIDEr\right)$$

## Analysis of experimental results

### Comparative experiments with feature extractors

**Fig. 10:**
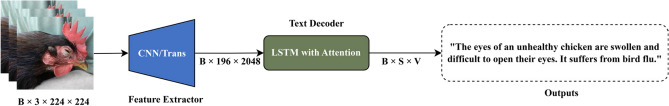
Comparative Experimental Framework Diagram.

In order to evaluate and explore the capability of various classical algorithm variants of convolutional neural networks (CNNs) and transformers in extracting high-level semantic information from images of poultry states, this study performs a series of comparative experiments based on the convolutional neural network with long short-term memory (CNN-LSTM) framework shown in Fig. 10. The primary aim of these experiments was to determine the most effective and suitable feature extractor for the extracting features from poultry states images.

Table 3 showed the performance of various models as feature extractors in extracting image features, including ResNet50, ResNet101^30^, ResNeXt50^31^, ShuffleNetV2^32^, Vision Transformer^33^, EfficientNet^34^, RegNet^35^, and Swin Transformer^36^, among others. These extracted features are subsequently processed by an LSTM^37^ network with an integrated attention mechanism to generate the corresponding subtitles (Table 3).

**Table 3:** Evaluation Scores of Different CNN/Transformers on PSC-Captions.

Models	B1	B2	B3	B4	R1	R2	RL	CIDEr	SPICE	Sm
CNN-LSTM										
ResNet50	0.704	0.584	0.491	0.415	0.714	0.445	0.697	2.672	0.522	1.171
ResNet101	0.709	0.590	0.498	0.423	0.718	0.448	0.702	2.634	0.527	1.164
ShuffleNet	0.701	0.581	0.488	0.411	0.723	0.455	0.708	2.688	0.535	1.182
Effb0	0.712	0.595	0.503	0.428	0.721	0.453	0.705	2.705	0.530	1.187
Effb2	0.710	0.592	0.500	0.425	0.721	0.453	0.706	2.679	0.529	1.179
Effb7	0.703	0.584	0.491	0.414	0.714	0.441	0.698	2.597	0.522	1.150
RegNetX16	0.708	0.585	0.492	0.415	0.718	0.447	0.704	2.637	0.525	1.164
ResNeXt50	0.709	0.589	0.495	0.418	0.721	0.448	0.705	2.635	0.529	1.165
RXt101	0.712	0.595	0.503	0.428	0.722	0.454	0.707	2.724	0.530	**1.193**
Transformer-LSTM										
vit_b32	0.696	0.575	0.480	0.404	0.683	0.407	0.668	2.342	0.489	1.057
vit_b16	0.690	0.567	0.471	0.395	0.690	0.418	0.674	2.418	0.498	1.082
SwinTran	0.711	0.592	0.501	0.422	0.691	0.420	0.675	2.453	0.495	1.095

ResNeXt101 demonstrated the best overall performance, likely due to its incorporation of "cardinality," which improves the network’s expressiveness and computational efficiency by increasing the number of parallel paths. This design enhances ResNeXt101’s ability to capture and process complex image features. Thus, the number of paths to effectively increase the expressive power and computational efficiency of the network. By introducing the concept of “cardinality” thus, the number of paths improves the expressiveness and computational efficiency of the network. This design allows ResNeXt101 to effectively capture and process complex image features, making it particularly well-suited for extracting features from poultry state images.

### Comparative experiments with classical image caption models

Nine representative models were selected for comparison in this set of experiments, including LSTM and Transformer based methods, and all parameters are consistent for fair comparison. The model results and profiles are as follows:

The ShowTell model^16^ is a classic approach in the field of image caption generation that uses a basic CNN + LSTM combination to accomplish the task. Spitial model^38^ This model uses a Convolutional Neural Network (CNN) to extract the image features, and then selects the image region that is most relevant to the currently generated word through the attention mechanism. The FC model^39^ uses a CNN to encode the image, and inputs the LSTM as the first word to predict the next word. The Att2in model^39^ dynamically computes the weights of different regions of the image for the generated word based on the FC model. The adaptive model ^40^introduces the concept of “visual sentinels” that help the model determine when to pay more attention to verbal and visual images. The Att2all model ^41^ equips the model with an attention module and uses the attended image features as inputs to all LSTM gates. The AoANet model ^42^ proposes an attention module to compute the correlation between the features extracted from each attention block and the query. The Gridmodel^43^ proposes a grid-based attention model. The model divides images into grids and applies an attention mechanism to focus on different parts of the grid to generate more accurate and detailed captions. The M2 model^44^ proposes a Meshed-Memory Transformer to improve the representation of contextual information in image caption generation.

The results are shown in Table 4, which reports the results of each classical model on the commonly used evaluation metrics. With the same parameter settings, the Poultrans proposed in this paper achieves the best performance on all the metrics. In particular, it achieves 4.927 on the CIDEr metric, which is 2.16 higher than the best performing AoANet model. It was also confirmed that the status reports generated by Poultrans were easy to interpret.

**Table 4:** Results of the comparison experiments for the PoulTrans model and classical image caption methods.

Method	Bleu1	Bleu2	Bleu3	Bleu4	Rouge1	Rouge2	RougeL	CIDER	SPICE	Sm
ShowTell	0.700	0.580	0.487	0.414	0.721	0.456	0.705	2.708	0.535	1.187
Spitial	0.683	0.559	0.464	0.397	0.706	0.431	0.700	2.492	0.513	1.115
FC	0.707	0.587	0.492	0.415	0.691	0.424	0.690	2.389	0.455	1.070
Att2in	0.707	0.586	0.494	0.420	0.707	0.435	0.694	2.527	0.518	1.128
Att2all	0.687	0.565	0.471	0.400	0.714	0.447	0.701	2.627	0.524	1.158
AoANet	0.700	0.580	0.486	0.413	0.722	0.458	0.707	2.767	0.536	1.205
Gridmodel	0.707	0.587	0.493	0.416	0.723	0.457	0.709	2.737	0.536	1.195
M2	0.702	0.583	0.491	0.417	0.713	0.449	0.699	2.681	0.530	1.177
**PoulTrans**	**0.798**	**0.678**	**0.580**	**0.501**	**0.842**	**0.628**	**0.803**	**4.927**	**0.608**	**1.882**

Bold text represents the best performing indicator.

### Ablation experiments

In this section, we detail the key design decisions of the proposed PoulTrans model and provide a comprehensive analysis of the performance of its core components—including the ResNeXt101 image feature extractor, the CSA_Encoder (Multi-attention Feature Differentiation Encoder), the CSMTransformer text decoder, and the PS-Loss loss function—are comprehensively analyzed for their performance on five important evaluation metrics: BLEU-4, Rouge-L, CIDEr, SPICE and Sm. For the baseline experiments, after pre-training on the ImageNet dataset, a baseline training approach was designed using an image encoder with ResNet50 (and a text decoder with LSTM) in a similar configuration. To ensure fair and comparable experimental results and a better transition to CSMT, we used ResNeXt101 pre-trained on the ImageNet dataset as the image feature extractor and chose the 2-layer transformer as the text decoder in the third set of experiments. In this way, we aim to fairly compare the performance of image feature extractors and decoders of different architectures to further highlight the advantages and effectiveness of the PoulTrans model.

The results are shown in Table 5. Following the initial introduction of the ResNeXt101 image feature extractor, the model exhibits a notable enhancement in all evaluation metrics in comparison to the baseline model. This outcome substantiates the efficacy of ResNeXt101 in capturing the essential semantic features of images. The combination of the Transformer text decoder with the model results in a further improvement in performance, particularly in the CIDEr and Sm metrics. This demonstrates Transformer’s superior ability to process sequence data and generate text descriptions.

**Table 5:** Results of the ablation experiments for the PoulTrans model.

RXT101	Trans	CSA	CSMT	n	PS-Loss	B4	RL	CIDEr	Spice	${S}_{m}$
×	×	×	×	×	×	0.415	0.697	2.672	0.522	1.171
✓	×	×	×	×	×	0.428	0.707	2.724	0.530	1.193
✓	✓	×	×	×	×	0.429	0.752	4.071	0.539	1.593
✓	✓	C	×	×	×	0.434	0.760	4.157	0.551	1.624
✓	✓	S	×	×	×	0.442	0.765	4.461	0.562	1.717
✓	✓	✓	×	×	×	0.448	0.769	4.474	0.568	1.724
✓	✓	✓	✓	10	×	0.454	0.774	4.533	0.571	1.744
✓	✓	✓	✓	20	×	0.454	0.781	4.572	0.574	1.758
✓	✓	✓	✓	30	×	0.463	0.784	4.651	0.575	1.783
✓	✓	✓	✓	40	×	0.464	0.785	4.662	0.584	1.789
✓	✓	✓	✓	50	×	0.456	0.780	4.573	0.575	1.759
✓	✓	✓	✓	40	0.3:0.7	0.472	0.789	4.791	0.587	1.829
✓	✓	✓	✓	40	**0.5:0.5**	**0.501**	**0.803**	**4.927**	**0.608**	**1.882**
✓	✓	✓	✓	40	0.7:03	0.494	0.798	4.917	0.598	1.874

The underlined number represents the number of memory cells introduced and bold text represents the best performing indicator.

With the introduction of the CSA-Encoder, the model showed significant performance improvements across all evaluation metrics, regardless of whether channel attention or spatial attention was applied individually. This enhancement was even more pronounced when both attention mechanisms were combined. This result highlights the crucial role of the multi-attention mechanism in improving the model’s ability to focus on image details and enhance the precision of state descriptions. Notably, when the number of memory units was set to 40, the model achieved the highest performance on all evaluation metrics, demonstrating the importance of dynamic memory units in capturing long-term dependencies and deepening the model’s understanding of complex image content.

Furthermore, integrating the PS-Loss comparative learning loss function led to a further significant boost in the model’s performance across all evaluation metrics. This improvement was especially noticeable when the loss weight ratio was set to 0.5:0.5, where the BLEU-4, Rouge-L, and CIDEr metrics reached their highest values. These findings confirm the effectiveness of PS-Loss in optimizing the model’s learning process and strengthening the fusion capabilities between image and text modalities.

### Qualitative analysis

**Fig. 11:**
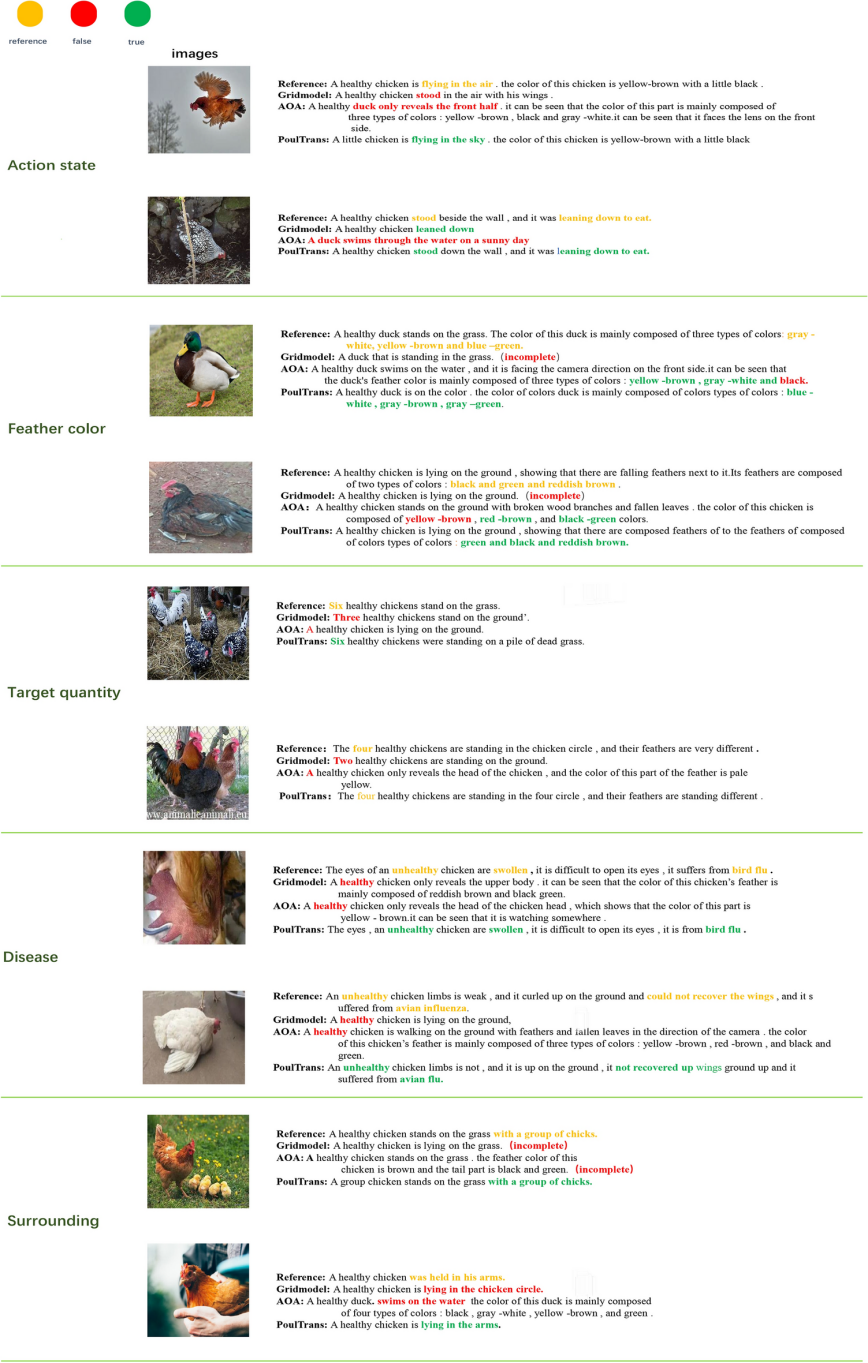
Sample Comparison of AOA, Gridmodel and PoulTrans.

In order to move beyond the reliance on evaluation metric scores to assess the quality of model-generated status reports, this study employed a qualitative approach to analysis. A random sample of images from the validation set, exhibiting notable differences in visual style and descriptive focus, was selected for analysis. The PoulTrans model and other classical captioning models were used to predict the captions of these images, with the results displayed in Fig. 11.

In the reference description, elements that require special attention are highlighted in orange; conversely, in the model’s predicted output, red indicates words that are incorrectly predicted, while green indicates words that are correctly predicted or that are semantically similar to the reference description.

The results demonstrate that the PoulTrans model exhibits superior performance compared to other models, particularly in the recognition of crucial health status information. Our model not only accurately predicts the category of poultry diseases from images of diseased poultry but also indicates the specific parts of the birds’ bodies that are the basis for disease determination. This represents a significant advancement in the field of poultry disease management, facilitating the creation of targeted treatments and preventive measures that are tailored to the specific site of pathology.

### Visualization analysis

**Fig. 12:**
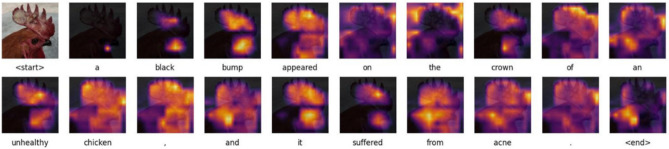
Heatmap visualization of image attention along the subtitle generation process.

In order to evaluate the effectiveness of the PoulTrans model in a targeted manner, an image of a poultry disease state was randomly sampled from the validation set. This image is presented in Fig. 12, which also includes a heat map of the attention on the image and a visualization of the caption generation process. It was observed that when the model generated the terms “black”, “bump”, “brown”, “chicken” and “sick”, it was unable to generate the captions. The attentional mechanism is able to correctly focus on the corresponding object or body region in the image when the terms “black”, “bump”, “crown”, “chicken”, and “acne” are generated. In particular, when generating the term “crown”, the model was able to accurately localize the crown of the bird. Furthermore, when generating descriptive words such as “suffered” and “appeared”, the model mainly focused on the relevant regions of the poultry head. This observation confirms the power of our model in capturing the correlation between poultry state features and appearance features, thereby validating the superiority of the model.

### Generalization experiments

**Fig. 13:**
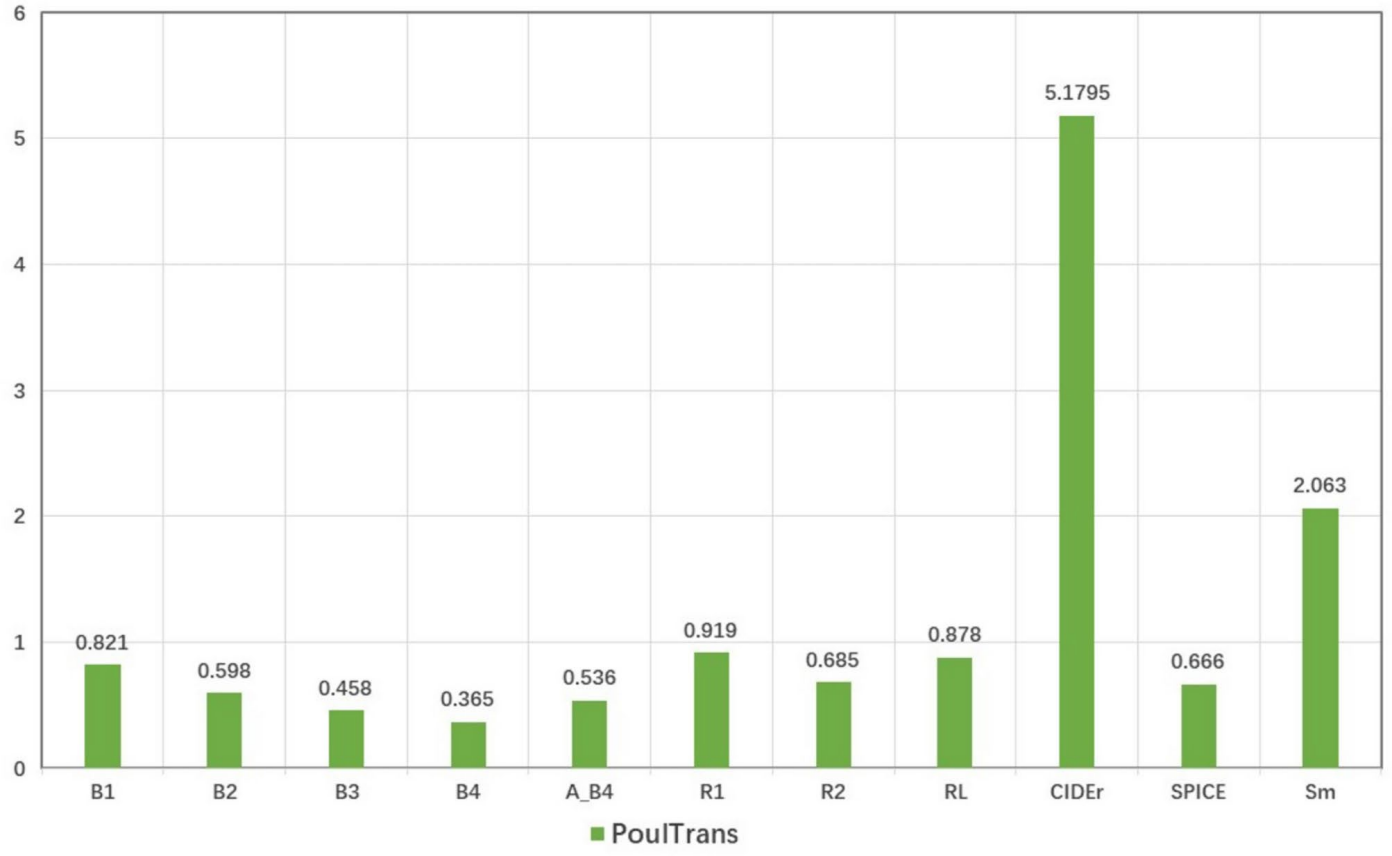
Performance of PoulTrans model on Flickr8k.

In this study, the model’s generalization ability was assessed using the Flickr8k dataset, a publicly available dataset of similar scale. Extensive testing revealed that the model achieved outstanding results across multiple evaluation metrics, as depicted illustrated in Fig. 13.

The evaluation on the Flickr8k dataset confirms the robustness and effectiveness of PoulTrans, which demonstrates its superior performance in multi-dimensional evaluation metrics. These findings further validate the applicability of the PSC-Captions dataset in enhancing model accuracy and reliability.

## Discussion

**Fig. 14:**
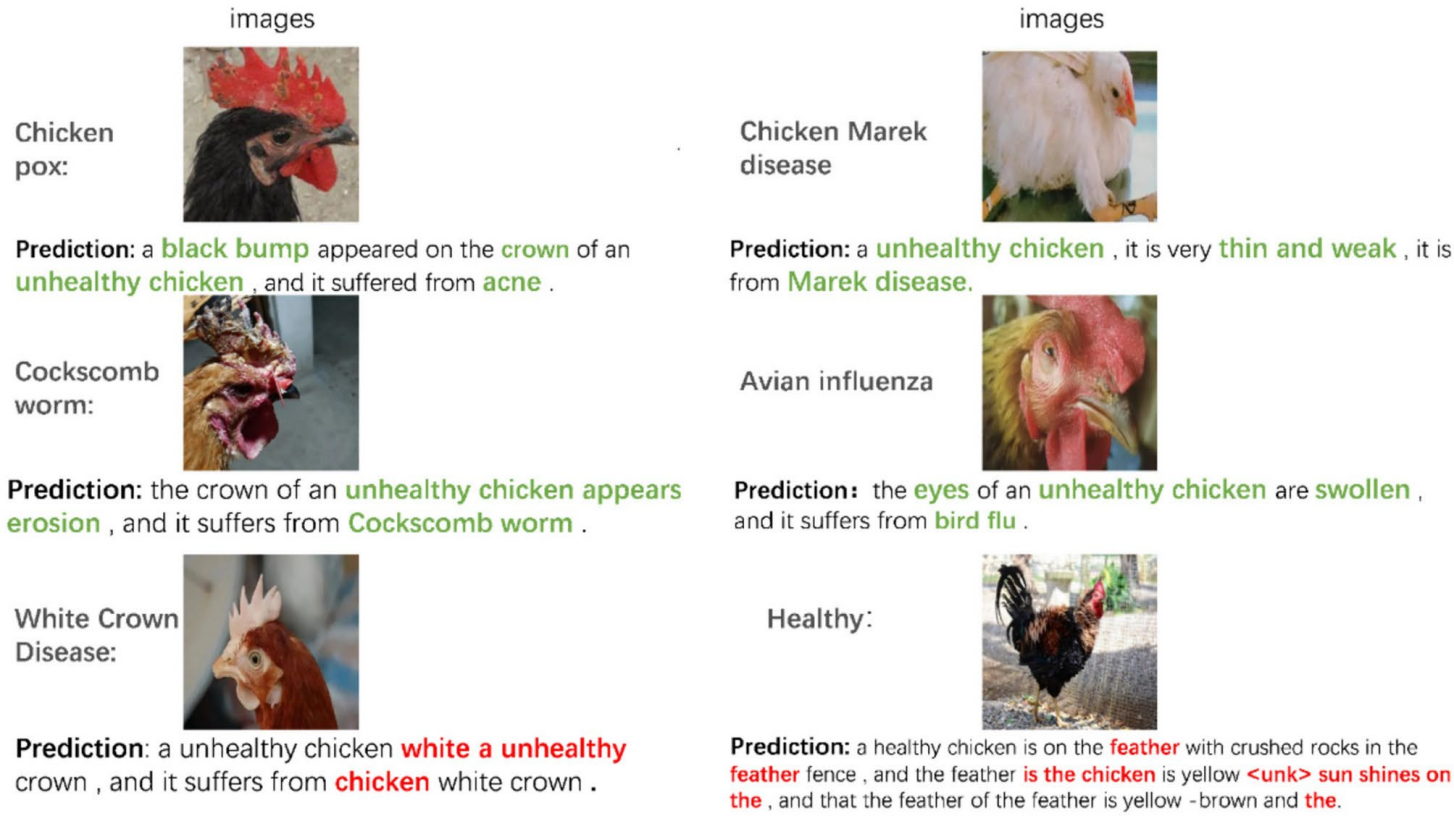
Examples of the performance of the PoulTrans model on different poultry diseases.

Although the validity of our proposed PoulTrans model was verified through metrics evaluation, qualitative analysis, and attention visualization, several key issues still require attention. We randomly interpreted the trained PoulTrans model, focusing on birds in an unhealthy state, as illustrated in Fig. 14. In this process, we identified several key issues.

The proposed model, PoulTrans, demonstrates efficacy in numerous domains, particularly in the prediction of poultry diseases such as chicken pox, chicken Marek’s disease, cockscomb ringworm, and poultry influenza. The table provides a visual representation of the model’s capacity to link images and text, with disease names highlighted in green bold font. Nevertheless, we observed shortcomings in the model’s predictions, such as jumbled words and semantic errors. These were particularly evident in instances where the model incorrectly identified healthy chickens with White Crown Disease, as evidenced by the presence of the < unk > tag in the descriptions of healthy chickens. To address this issue, an in-depth examination was conducted of the model’s architectural design and training process. It was determined that the issue originated from the threshold for word frequency established during the construction of the vocabulary list. The vocabulary list was compiled with a minimum word frequency of 2, excluding words that appeared only once. This resulted in the decoder using < unk > tags, which affected the accuracy and readability of the model output. This resulted in the decoder being unable to identify suitable words for use in descriptions, necessitating the use of < unk > tags. This had an adverse effect on the accuracy and readability of the model output. Reducing the word frequency threshold to 1 resulted in the inclusion of all words, but this approach led to a large vocabulary size and an increase in the number of errors made by the decoder in selecting words.

As the dataset expands and the description content becomes more detailed, it may become a significant research focus to address the challenge of balancing the dataset size and vocabulary capacity in order to effectively address these issues. While the poultrans model performs less effectively in a few samples, it accurately recognizes key terms such as “unhealthy”, “chicken”, disease names, disease characteristics, and the disease’s location. Consequently, the model makes few errors. We are optimistic about the future performance of PoulTrans and believe its performance can be improved through the following strategies:

1. Explore comprehensive loss functions that consider not only the deviation of image features from the semantic information but also introduce semantic weighting for feature-related words.
2. Find a balanced approach to expanding the dataset size and vocabulary capacity.
3. Continue to enrich the "PSC-Captions" dataset by expanding beyond the current four categories of poultry.

## Conclusion

Taken together in this study, we developed the PoulTrans model, a novel end-to-end trainable framework for poultry status report generation, and created the first image captioning dataset, PSC-Captions, specialized for poultry status description. The framework integrates the ResNeXt101 feature extractor, CSA-Encoder multi-attention encoder, and CSMTransformer multi-feature attention converter alongside the innovative PS-Loss function, which considers the bias loss between image features and semantic information. Based on this framework, the feature extractor captures visual multi-attention features, the multi-attention encoder outputs spatial channel attention for different lesion regions, and the decoder accurately learns and transcribes the knowledge embedded in the image content.

The PSC-Captions dataset construction provides a valuable benchmark for poultry status determination studies and positively impacts agricultural research overall. PoulTrans, as a specialized, end-to-end trainable framework, excels in generating poultry status descriptions and holds promise applications across various livestock categories. We believe that image captioning technology will play a key role in poultry farming and the agricultural industry. The generated natural language description was not only easy to understand but also enable farmers to quickly respond to any abnormal conditions in their birds. Moreover, this technology is anticipated to drive significant advancements in crop breeding as well.

## Data Availability

The data code is available online at: https://github.com/kong1107800/PoulTrans. Because the image data set occupies a large amount of storage, the datasets used and/or analyzed during the current study are available from the corresponding author upon reasonable request.
